# FOLFIRI as a second-line therapy in patients with docetaxel-pretreated gastric cancer: a historical cohort

**DOI:** 10.1186/1756-9966-32-67

**Published:** 2013-09-17

**Authors:** Marcello Maugeri-Saccà, Laura Pizzuti, Domenico Sergi, Maddalena Barba, Franca Belli, Silvia Ileana Fattoruso, Diana Giannarelli, Antonella Amodio, Sara Boggia, Patrizia Vici, Luigi Di Lauro

**Affiliations:** 1 Division of Medical Oncology B, “Regina Elena” National Cancer Institute, Via Elio Chianesi, 53 00144 Rome, Italy; 2 Scientific Direction, “Regina Elena” National Cancer Institute, Via Elio Chianesi, 53 00144 Rome, Italy; 3 Division of Oncology, Spolverini Hospital, Ariccia, Italy; 4 Division of Oncology, Fiorini Hospital, Terracina, Italy; 5 Division of Biostatistics, “Regina Elena” National Cancer Institute, Via Elio Chianesi, 53 00144 Rome, Italy; 6 Sant’Andrea Hospital, Via di Grottarossa, 1035 00189 Rome, Italy

**Keywords:** FOLFIRI, Gastric cancer, Second-line chemotherapy

## Abstract

**Background:**

The role of second-line therapy in gastric cancer patients mostly stemmed from clinical trials with monochemotherapy carried out in Asian countries. Nevertheless, these results cannot be broadly generalized as molecular studies suggested the existence of different sets of deregulated gene networks correlated with ethnicity. In the present study, we investigated the activity and safety of FOLFIRI given as a second-line therapy in metastatic gastric or gastro-esophageal junction cancer patients who experienced disease progression on or after first-line docetaxel-containing chemotherapy.

**Methods:**

Patients with histologically confirmed metastatic gastric cancer who failed docetaxel-containing first-line therapy and who received FOLFIRI in second line were eligible for the study. Seventy patients treated at three Italian cancer centers between 2005 and 2012 entered the study. Patients received every 2 weeks irinotecan 180 mg/m^2^ as 1 h infusion on day 1, folinic acid 100 mg/m^2^ intravenously days 1–2, and fluorouracil as a 400 mg/m^2^ bolus and then 600 mg/m^2^ continuous infusion over 22 hours days 1–2.

**Results:**

We observed 1(1.4%) complete response, 15 (21.4%) partial response, for an overall response rate of 22.8% (95% confidence interval (CI): 13.4-32.3). Stable disease was recorded in 21 (30%) patients. Median progression-free survival and overall survival were 3.8 months (95% CI: 3.3-4.4) and 6.2 months (95% CI: 5.3-7.1), respectively. The treatment was well tolerated, as the most common G3-4 toxicities were neutropenia (28.5%) and diarrhea (14.5%).

**Conclusions:**

FOLFIRI appears an effective and safe treatment option for pretreated metastatic gastric cancer patients, and deserves further investigation in randomized clinical trials.

## Background

Gastric cancer and cancer of the gastro-oesophageal junction (GEJ) are a significant global health problem, representing the fourth most common cancer diagnosed worldwide [1]. The prognosis for these patients remain poor, as the majority of them are diagnosed with locally advanced or metastatic disease with a median survival of 7–10 months [2].

Several randomized clinical trials demonstrated the role of chemotherapy in the first-line setting, as different regimens determined an improvement in survival and in quality of life (QoL) compared with best supportive care (BSC) alone [3-5]. More recently, a wave of randomized clinical trials with superiority design was successfully completed, and novel active drugs such as docetaxel [6], S1 [7] and trastuzumab [8] changed the landscape of the clinical management of gastric cancer. Other agents including capecitabine [9], oxaliplatin [10] and irinotecan [11] have proven antitumor activity, thus expanding the spectrum of therapeutic options available in the first-line setting. Even though novel active drugs and combinations entered the therapeutic scenario, second-line treatment has been historically considered largely empirical. Furthermore, geographic distributions exist in chemotherapy administration beyond first-line, being prevalently adopted in Asian countries. Indeed, the rates of administration of subsequent chemotherapy significantly differed among phase III studies conducted in front-line, spanning from 14% in the UK REAL 2 study [9] to 75% in the Japanese SPIRITS trial [7].

The clinical proof-of-concept for second-line chemotherapy stemmed from two recent randomized phase III trials, demonstrating the superiority of second-line monotherapy (docetaxel or irinotecan) over BSC [12,13]. Nevertheless, it is foreseeable that a widespread adoption of second-line chemotherapy will further be limited by multiple factors. Firstly, the non-Asian study was prematurely closed when only one-third of the preplanned 120 patients were enrolled [12]. As a result, evidence supporting second-line chemotherapy in non-Asian patients are still scattered being mostly extrapolated from the Korean study. Secondly, the different biological background of gastric cancer arising in Asian and Western patients must be taken into account as a potential confounding factor [14]. Finally, single-agent therapy may result suboptimal, at least for patients with good performance status.

On this basis, we conducted a retrospective study in order to evaluate the activity and safety of FOLFIRI given as a second-line therapy in a cohort of docetaxel-pretreated metastatic gastric cancer patients.

## Methods

The study population was composed by patients with metastatic gastric or GEJ cancer who experienced disease progression on or after first-line docetaxel-containing chemotherapy. Patients were treated at three Italian cancer centers between 2005 and 2012. The majority of patients was selected from the “Regina Elena” National Cancer Institute, Rome. Medical records were reviewed in order to obtain information on demography, treatment received, safety and outcomes.

Patients with histologically confirmed, docetaxel-pretreated metastatic gastric cancer who received FOLFIRI in second line were eligible for the study. Other eligibility criteria included Eastern Cooperative Oncology Group performance status ≤2 (ECOG PS), aged between 18 and 75 years, adequate bone marrow (absolute neutrophil count ≥1500/μl, platelet count ≥100 000/μl), renal (serum creatinine ≤1.5 mg/dl) and liver (serum bilirubin ≤2 mg/dL) functions, normal cardiac function, absence of second primary tumor other than non-melanoma skin cancer or in situ cervical carcinoma, no central nervous system involvement, no prior radiotherapy in target lesions, and no concurrent uncontrolled medical illness.

Patients received every 2 weeks irinotecan 180 mg/m^2^ as 1 h infusion on day 1, folinic acid 100 mg/m^2^ intravenously days 1–2, and fluorouracil as a 400 mg/m^2^ bolus and then 600 mg/m^2^ continuous infusion over 22 hours days 1–2. The dose of irinotecan was reduced to 150 mg/m^2^ in patients older than 70 years. Chemotherapy was generally administered on an outpatient basis for a maximum of 12 cycles. Treatment was continued until disease progression or unacceptable toxicity.

Toxicity was graded according to the National Cancer Institute-Common Toxicity Criteria version 4.0 (NCI-CTC v. 4.0). Tumor response was evaluated according to the response evaluation criteria for solid tumors (RECIST 1.1). Progression-free survival (PFS) and overall survival (OS) were calculated from the date of therapy initiation to the date of disease progression, death from any cause or last follow-up evaluation, respectively. PFS and OS were analyzed according to the Kaplan-Meier method. The Cox proportional hazards regression model was used for univariate analysis of prognostic factors for survival. All statistical analyses were performed using SPSS statistical software version 20 (SPSS inc.,Chicago IL, USA). The study was approved by the coordinating centre’s Ethics Committee at the “Regina Elena” National Cancer Institute, Rome, and was carried out according to the principles of the Declaration of Helsinki. A written informed consent was obtained from all patients.

## Results

### Patients characteristics

Seventy patients with a median age of 65 years (range, 32–75) were included in this study. Patients’ characteristics are illustrated in Table 1. The primary tumor site was stomach in 54 patients (77%) and the GEJ in 16 patients (23%). The histology subtype was diffuse, intestinal and unknown in 33 (47%), 29 (41.5%), and 8 (11.5) patients, respectively. Primary tumor resection was carried out in twenty-five patients (36%). The ECOG PS was 0, 1 and 2 in 10 (14.5%), 40 (57%) and 20 (28.5%) patients, respectively. Fifty-three patients (76%) had two or more metastatic sites. PFS during first-line chemotherapy was ≥ 6 months in 42 patients (60%), and the chemotherapy-free interval was > 3 months in 38 patients (54%). Among regimens administered in the first-line setting, 25 patients (36%) received docetaxel, oxaliplatin and capecitabine [15], 20 patients (28.5%) received epirubicin, cisplatin and docetaxel [16], 19 patients (27%) were treated with epirubicin, oxaliplatin and docetaxel [17], and 6 patients (8.5%) received cisplatin and docetaxel [18].

**Table 1:** Patient characteristics

**Characteristic**	**No. of patients**	**%**
**Patients evaluable**	70	100
**Age, years**		
Median (range)	65 (32–75)	
**Sex**		
Male	41	58.5
Female	29	41.5
**Response to prior chemotherapy**		
Yes	44	63
No	26	37
**Status of primary tumor**		
Resected	25	36
Unresected	45	64
**Tumor histology**		
Diffuse	33	47.2
Intestinal	29	41.4
Unknown	8	11.4
**ECOG PS**		
0	10	14.5
1	40	57
2	20	28.5
**Number of metastatic sites**		
1	17	24
2	32	46
3	21	30
**Site of metastases**		
Liver	48	68.5
Nodes	41	58.5
Peritoneum	41	58.5
Lung	13	18.5
Bone	6	8.5
**PFS under first-line chemotherapy**		
≥ 6 months	42	60
< 6 months	28	40
**Chemotherapy-free interval**		
> 3 months	38	54
< 3 months	32	46

*Abbreviations*: ECOG PS Eastern Cooperative Oncology Group Performance Status.

### Efficacy

**Figure 1:**
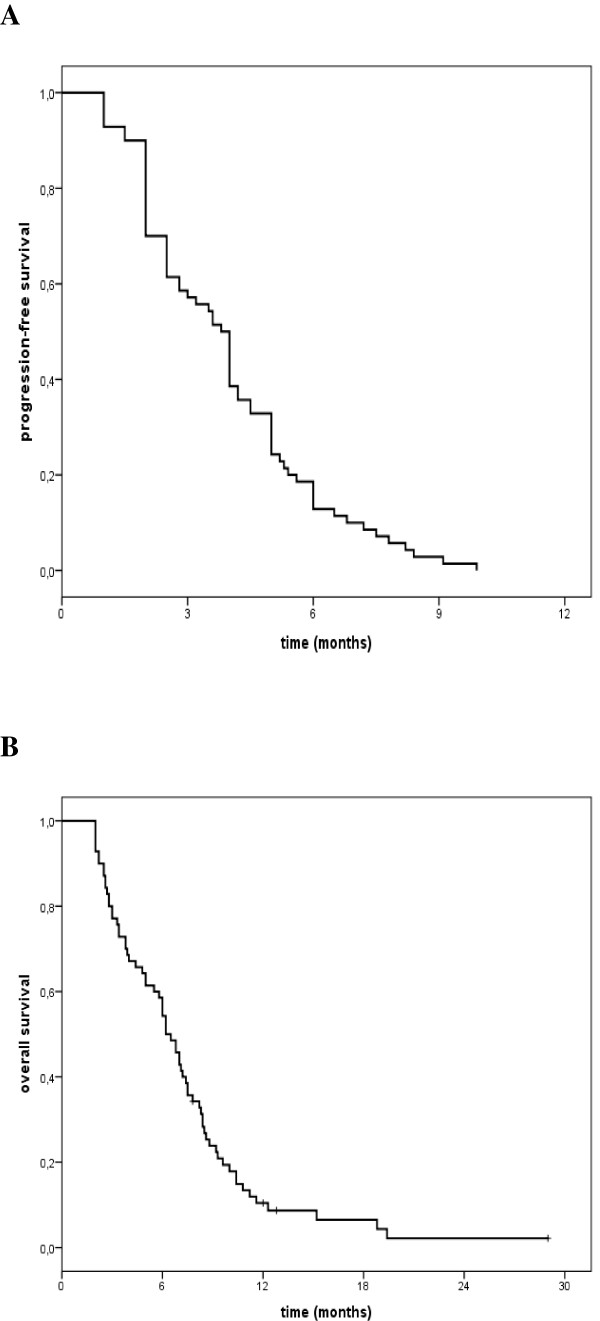
**Kaplan–Meier curves. (A)** progression-free survival. **(B)** overall survival.

Response to treatment is illustrated in Table 2. Among 70 assessable patients, we observed 1(1.4%) complete response (CR), 15 (21.4%) partial responses (PR), for an overall response rate (ORR) of 22.8% (95% confidence interval (CI): 13.4-32.3). Stable disease (SD) was recorded in 21 (30%) patients, translating into a disease control rate (DCR) of 52.8%. Median PFS was 3.8 months (95% CI: 3.3-4.4), and median OS was 6.2 months (95% CI: 5.3-7.1) (Figure 1). In univariate analysis, the only significant predictors of OS were ECOG PS (0–1 vs 2: 7.0 months [95% CI: 5.7-8.3] vs 5.0 months [95%CI: 2.4-7.6], *P =* 0.01; HR 1.94 [95% CI: 1.13-3-33]) and PFS under first-line chemotherapy (≥ 6 months vs < 6 months: 7.1 months [95% CI: 6.2-8.0] vs 4.0 months [95% CI: 2.7-5.3], p = 0.04; HR 1.67 [95% CI: 1.02-2.34]). We did not observe any significant difference in efficacy nor in PFS and OS between patients who received fluoropyrimidine in the first-line compared with patients who did not (ORR: 24.4% vs 20%; PFS 3.8 vs 4.0 months, *P =* 0.79; OS 6.2 vs 6.5 months, *P =* 0.61).

**Table 2:** Response rate in 70 patients

**Responses**	**No. of patients**	**%**
Complete response	1	1.4
Partial response	15	21.4
Stable disease	21	30
Progressive disease	33	47.2

### Toxicity

Toxicities are listed in Table 3. A total of 352 cycles of FOLFIRI were analyzed in 70 patients, with a median of 6 cycles administered per patient (range, 2–12). The most common G3-4 toxicities were neutropenia (28.5%) and diarrhea (14.5%). Treatment discontinuation was necessary in 4 patients (5.7%). A 50% dose reduction was required in 2 patients (2.8%) for recurrent G3 diarrhea, whereas a 25% dose reduction was needed in 11 patients (21.2%), mostly correlated with G3 diarrhea (7 patients). Five patients required granulocyte colony-stimulating factor (G-CSF) for G4 neutropenia.

**Table 3:** Main toxicity in 70 patients

**Toxicity**	**Grade 3 (%)**	**Grade 4 (%)**
Neutropenia^a^	21.5	7
Anemia	7	-
Thrombocytopenia	3	-
Diarrhea	13	1.4
Nausea/vomiting	6	-
Mucositis	6	-
Fatigue	6	-
Hepatotoxicity	3	-

^a^Four episodes of febrile neutropenia in 3 patients (4%).

## Discussion

In the present study, we reported that the use of FOLFIRI in the second-line setting in docetaxel-pretreated metastatic gastric cancer is associated with an ORR of 22.8% and a DCR of 52.8%. Median PFS and OS were 3.8 months and 6.2 months, respectively. To our knowledge, this is one of the largest series presented so far with second-line chemotherapy combination in non-Asian patients.

In the second-line setting, only two recent studies exploring the benefit of palliative chemotherapy were presented in full text. The Arbeitsgemeinschaft Internistische Onkologie (AIO) conducted in Germany analyzed single agent irinotecan (250 mg/m^2^ every 3 weeks, increased to 350 mg/m^2^ after the first cycle depending on toxicity) versus BSC [12]. Primary endpoint was OS. Even though the hazard ratio for death was 0.48 (95% CI 0.25–0.92), results must be interpreted with caution. Only 40 patients of the preplanned 120 entered the study, which closed prematurely due to poor accrual. Regarding efficacy, no objective tumor responses were documented, and disease stabilization for at least 6 weeks was reported in 53% of patients. We are aware of the intrinsic limitations of both retrospective studies and indirect comparisons. In our study, patient characteristics were similar, with the exception that in the AIO study none of the patients allocated in the irinotecan arm received docetaxel in first-line. However, even though the DCR was similar (52.8% *vs* 53%), we reported an ORR of 22.8%. Apparently, FOLFIRI compares favorably when considering PFS (3.8 months *vs* 2.5 months) and OS (6.2 months vs 4.0 months). Surprisingly, FOLFIRI seemed to be better tolerated than irinotecan monotherapy (G3-4 diarrhea 14.4% vs 26%, neutropenic fever 4% vs 16%), probably because of the lower irinotecan cumulative dose and the different schedule.

In the second phase III trial, 202 Korean patients were randomized in a 2:1 fashion to receive either chemotherapy, consisting in biweekly irinotecan 150 mg/m^2^ or docetaxel 60 mg/m^2^ every 3 weeks at the physician’s discretion, or BSC [13]. Docetaxel-containing chemotherapy was administered only in the 3% of patients. The intention to treat analysis showed an increase in OS with chemotherapy (5.3 months vs 3.8 months) with a HR of 0.657 (95% CI: 0.485-0.891, *P =* 0.007). No differences were seen in correlation with the type of chemotherapeutic agent, thus complementing the results from the Japanese phase III WJOG4007 study (reported only in abstract form) and from an European, randomized, three-arm phase II study which also evaluated a liposomal nanocarrier formulation of irinotecan [19,20]. Even though these results have to be considered as a major step forward in the management of gastric cancer, we believe they cannot be broadly generalized. It is known that the topographic distribution (distal vs proximal), pathological features (intestinal vs diffuse) and, even more importantly, survival outcome differ between Asian and Western patients [14,21,22]. Treatment pattern is one of the factors proposed to explain such discrepancies, as extensive D2 resection in early stage gastric cancer is routinely used in Asian countries. Nevertheless, a comparison of surgical outcomes between patients treated at the Memorial Sloan Kettering Cancer Center, where D2 resection is extensively carried out, and patients treated in Korea revealed better disease-specific survival for the latter group [23]. Therefore, it is foreseeable that underlying biological differences play a crucial role, and growing evidence indicate that the molecular taxonomy of gastric cancer is influenced by ethnic factors. MicroRNA expression profiling, which is emerging as an excellent classifier in oncology, and next-generation sequencing studies are beginning to unveil the existence of different sets of deregulated gene networks potentially correlated with ethnicity [24-26]. Furthermore, the molecular analysis of the ToGA trial revealed that HER2 positivity is associated with the intestinal-type gastric cancer (32.5% intestinal vs 6.0% diffuse), the most common histology in Asia [8]. Overall, the different ethnicity-related molecular landscape of gastric cancer might reflect a different expression of therapeutic targets and, in turn, sensitivity to anticancer agents. Beyond tumor biology, also pharmacogenomic differences should be taken into account. For instance, while S1 is extensively used in front-line in Asia, its use in the Western hemisphere was initially constrained by evidence of more severe toxicity in Caucasian patients [27]. The different magnitude of toxic effects is thought to be correlated with CYP2A6 gene polymorphisms, affecting the conversion of S1 to fluorouracil. Indeed, in the phase III FLAG study conducted in non-Asian countries S1 was used at a lower dose compared to Japanese studies [28], despite the higher body surface of Western patients.

Next, in the European FFCD-GERCOR-FNCLCC trial 416 patients were randomized to receive two different sequential strategies in first- and second-line: epirubicin, cisplatin and capecitabine in first-line and FOLFIRI in second-line vs the reverse sequence [29]. The sequence with FOLFIRI in first-line resulted superior for the primary endpoint (time to treatment failure), a benefit deriving from the better tolerance and the correlated lower rate of treatment discontinuation. However, no firm conclusions can be drawn from this trial having been only presented in abstract form to date.

Finally, a recent retrospective Turkish study reported data from 97 docetaxel-pretreated patients who received FOLFIRI in the second-line setting [30]. Investigators reported an ORR of 26.8% and a DCR of 58.8%. However, it is worth considering that 19 patients (19.5%) had locally recurrent gastric cancer and 47 patients (48.5%) had only one metastatic site. In our opinion, the rather heterogeneous study cohort along with the inclusion of a consistent fraction of patients with lower tumor burden compared to those examined in our study led to an overestimation of the results, as investigators reported a median OS of 10.5 months.

## Conclusions

FOLFIRI appears an effective and safe treatment option for pretreated metastatic gastric cancer patients. However, second-line chemotherapy comparative trials are needed to better define the role of FOLFIRI in gastric cancer (e.g. versus monochemotherapy).

## Competing interests

The authors declared that they have no competing interests.

## Authors’ contributions

LDL and MM-S conceived and designed the study, LP, DS, MB, FB, SIF, AA, SB and PV collected and assembled the data, DG performed the statistical analysis, MM-S and LDL wrote the manuscript. All authors read and approved the final manuscript.

## References

[B1] Jemal A Bray F Center MM Ferlay J Ward E Forman D Global cancer statistics CA Cancer J Clin 2011 61 69 90 10.3322/caac.20107 21296855

[B2] Cervantes A Roda D Tarazona N Roselló S Pérez-Fidalgo JA Current questions for the treatment of advanced gastric cancer Cancer Treat Rev 2013 39 60 67 10.1016/j.ctrv.2012.09.007 23102520

[B3] Glimelius B Ekström K Hoffman K Graf W Sjödén PO Haglund U Svensson C Enander LK Linné T Sellström H Heuman R Randomized comparison between chemotherapy plus best supportive care with best supportive care in advanced gastric cancer Ann Oncol 1997 8 163 168 10.1023/A:1008243606668 9093725

[B4] Murad AM Santiago FF Petroianu A Rocha PR Rodrigues MA Rausch M Modified therapy with 5-fluorouracil, doxorubicin, and methotrexate in advanced gastric cancer Cancer 1993 72 37 41 10.1002/1097-0142(19930701)72:1<37::AID-CNCR2820720109>3.0.CO;2-P 8508427

[B5] Pyrhönen S Kuitunen T Nyandoto P Kouri M Randomised comparison of fluorouracil, epidoxorubicin and methotrexate (FEMTX) plus supportive care with supportive care alone in patients with non-resectable gastric cancer Br J Cancer 1995 71 587 591 10.1038/bjc.1995.114 7533517 PMC2033628

[B6] Van Cutsem E Moiseyenko VM Tjulandin S Majlis A Constenla M Boni C Rodrigues A Fodor M Chao Y Voznyi E Risse ML Ajani JA V325 Study Group. Phase III study of docetaxel and cisplatin plus fluorouracil compared with cisplatin and fluorouracil as first-line therapy for advanced gastric cancer: a report of the V325 Study Group J Clin Oncol 2006 24 4991 4997 10.1200/JCO.2006.06.8429 17075117

[B7] Koizumi W Narahara H Hara T Takagane A Akiya T Takagi M Miyashita K Nishizaki T Kobayashi O Takiyama W Toh Y Nagaie T Takagi S Yamamura Y Yanaoka K Orita H Takeuchi M S-1 plus cisplatin versus S-1 alone for first-line treatment of advanced gastric cancer (SPIRITS trial): a phase III trial Lancet Oncol 2008 9 215 221 10.1016/S1470-2045(08)70035-4 18282805

[B8] Bang YJ Van Cutsem E Feyereislova A Chung HC Shen L Sawaki A Lordick F Ohtsu A Omuro Y Satoh T Aprile G Kulikov E Hill J Lehle M Rüschoff J Kang YK ToGA Trial Investigators Trastuzumab in combination with chemotherapy versus chemotherapy alone for treatment of HER2-positive advanced gastric or gastro-oesophageal junction cancer (ToGA): a phase 3, open-label, randomised controlled trial Lancet 2010 376 687 697 10.1016/S0140-6736(10)61121-X 20728210

[B9] Cunningham D Starling N Rao S Iveson T Nicolson M Coxon F Middleton G Daniel F Oates J Norman AR Upper Gastrointestinal Clinical Studies Group of the National Cancer Research Institute of the United Kingdom Capecitabine and oxaliplatin for advanced esofagogastric cancer N Eng J Med 2008 358 36 46 10.1056/NEJMoa073149 18172173

[B10] Al-Batran SE Hartmann JT Probst S Schmalenberg H Hollerbach S Hofheinz R Rethwisch V Seipelt G Homann N Wilhelm G Schuch G Stoehlmacher J Derigs HG Hegewisch-Becker S Grossmann J Pauligk C Atmaca A Bokemeyer C Knuth A Jäger E Phase III trial in metastatic gastroesophageal adenocarcinoma with fluouracil, leucovorin plus either oxaliplatin or cisplatin: a study of the arbeitgemeinschaft internistische onkologie J Clin Oncol 2008 26 1435 1442 10.1200/JCO.2007.13.9378 18349393

[B11] Bouché O Raoul JL Bonnetain F Giovannini M Etienne PL Lledo G Arsène D Paitel JF Guérin-Meyer V Mitry E Buecher B Kaminsky MC Seitz JF Rougier P Bedenne L Milan C Randomized multicenter phase II trial of a biweekly regimen of fluouracil and leucovorin (LV5FU2), LV5FU2 plus cisplatin, or LV5FU2 plus irinotecan in patients with previously untreated metastatic gastric cancer: a Fédération Francophone de Cáncerologie Digestive Group Study – FFCD 9803 J Clin Oncol 2004 22 4319 4328 10.1200/JCO.2004.01.140 15514373

[B12] Thuss-Patience P Kretzschmar A Bichev D Deist T Hinke A Breithaupt K Dogan Y Gebauer B Schumacher G Reichardt P Survival advantage for irinotecan versus best supportive care as second-line chemotherapy in gastric cancer - a randomized phase III study of the Arbeitgemeinschaft Internische Onkologie (AIO*)* Eur J Cancer 2011 15 2306 2314 21742485 10.1016/j.ejca.2011.06.002

[B13] Kang JH Lee SI Lim DH Park KW Oh SY Kwon HC Hwang IG Lee SC Nam E Shin DB Lee J Park JO Park YS Lim HY Kang WK Park SH Salvage chemotherapy for pretreated gastric cancer: a randomized phase III trial comparing chemotherapy plus best supportive care with best supportive care alone J Clin Oncol 2012 30 1513 1518 10.1200/JCO.2011.39.4585 22412140

[B14] Kim R Tan A Choi M El-Rayes BF Geographic differences in approach to advanced gastric cancer: Is there a standard approach? Crit Rev Oncol Hematol 2013 doi: 10.1016/j.critrevonc.2013.05.007. [Epub ahead of print] 10.1016/j.critrevonc.2013.05.007 23764501

[B15] Di Lauro L Sergi D Belli F Fattoruso SI Arena MG Pizzuti L Vici P Docetaxel, oxaliplatin, and capecitabine (DOX) combination chemotherapy for metastatic gastric or gastroesophageal junction (GEJ) adenocarcinoma [abstract ] J Clin Oncol 2013 31 Suppl e15065

[B16] Di Lauro L Belli F Arena MG Carpano S Paoletti G Giannarelli D Lopez M Epirubicin, cisplatin and docetaxel combination therapy for metastatic gastric cancer Ann Oncol 2005 16 1498 1502 10.1093/annonc/mdi281 15956036

[B17] Di Lauro L Giacinti L Arena MG Sergi D Fattoruso SI Giannarelli D Lopez M Phase II study of epirubicin, oxaliplatin and docetaxel combination in metastatic gastric or gastroesophageal junction adenocarcinoma J Exp Clin Cancer Res 2009 28 34 10.1186/1756-9966-28-34 19267943 PMC2657908

[B18] Roth AD Maibach R Martinelli G Fazio N Aapro MS Pagani O Morant R Borner MM Herrmann R Honegger H Cavalli F Alberto P Castiglione M Goldhirsch A Docetaxel (Taxotere)-cisplatin (TC): an effective drug combination in gastric carcinoma. Swiss Group for Clinical Cancer Research (SAKK), and the European Institute of Oncology (EIO) Ann Oncol 2000 11 301 306 10.1023/A:1008342013224 10811496

[B19] Ueda S Hironaka S Yasui H Nishina T Tsuda M Tsumura T Sugimoto N Shimodaira H Tokunaga S Moriwaki T Esaki T Nagase M Fujitani K Yamaguchi K Ura T Hamamoto Y Morita S Okamoto I Boku N Hyodo I Randomized phase III study or irinotecan (CPT-11) versus weekly paclitaxel (wPTX) fir advanced gastric cancer (AGC) refractory to combination chemotherapy (CT) of fluoropyrimidine plus platinum (FP): WJOG4007 [abstract ] J Clin Oncol 2012 30 Suppl 4002

[B20] Roy AC Park SR Cunningham D Kang YK Chao Y Chen LT Rees C Lim HY Tabernero J Ramos FJ Kujundzic M Cardic MB Yeh CG de Gramont A A randomized phase II study of PEP02 (MM-398), irinotecan or docetaxel as a second-line therapy in patients with locally advanced or metastatic gastric or gastro-oesophageal junction adenocarcinoma Ann Oncol 2013 24 1567 1573 10.1093/annonc/mdt002 23406728

[B21] Ito H Inoue H Ikeda H Onimaru M Yoshida A Hosoya T Sudo K Eleftheriadis N Maselli R Maeda C Wada Y Sando N Hamatani S Kudo SE Clinicopathological characteristics and treatment strategies in early gastric cancer: a retrospective cohort study J Exp Clin Cancer Res 2011 30 117 10.1186/1756-9966-30-117 22206626 PMC3339341

[B22] Ito H Inoue H Odaka N Satodate H Suzuki M Mukai S Takehara Y Kida H Kudo SE Clinicopathological characteristics and optimal management for esophagogastric junctional cancer; a single center retrospective cohort study J Exp Clin Cancer Res 2013 32 2 10.1186/1756-9966-32-2 23289488 PMC3560249

[B23] Strong VE Song KY Park CH Jacks LM Gonen M Shah M Coit DG Brennan MF Comparison of gastric cancer survival following R0 resection in the United States and Korea using an internationally validated nomogram Ann Surg 2010 251 640 646 10.1097/SLA.0b013e3181d3d29b 20224369

[B24] Volinia S Calin GA Liu CG Ambs S Cimmino A Petrocca F Visone R Iorio M Roldo C Ferracin M Prueitt RL Yanaihara N Lanza G Scarpa A Vecchione A Negrini M Harris CC Croce CM A microRNA expression signature of human solid tumors defines cancer gene targets Proc Natl Acad Sci U S A 2006 103 2257 2261 10.1073/pnas.0510565103 16461460 PMC1413718

[B25] Ueda T Volinia S Okumura H Shimizu M Taccioli C Rossi S Alder H Liu CG Oue N Yasui W Yoshida K Sasaki H Nomura S Seto Y Kaminishi M Calin GA Croce CM Relation between microRNA expression and progression and prognosis of gastric cancer: a microRNA expression analysis Lancet Oncol 2010 11 136 146 10.1016/S1470-2045(09)70343-2 20022810 PMC4299826

[B26] Liang H Kim YH Identifying molecular drivers of gastric cancer through next-generation sequencing Cancer Lettdoi: 10.1016/j.canlet.2012.11.029. [Epub ahead of print] 10.1016/j.canlet.2012.11.029 PMC3873853 23178814

[B27] Ajani JA Faust J Ikeda K Yao JC Anbe H Carr KL Houghton M Urrea P Phase I pharmacokinetic study of S-1plus cisplatin in patients with advanced gastric carcinoma J Clin Oncol 2005 23 6957 6965 10.1200/JCO.2005.01.917 16145066

[B28] Ajani JA Rodriguez W Bodoky G Moiseyenko V Lichinitser M Gorbunova V Vynnychenko I Garin A Lang I Falcon S Multicenter phase III comparison of cisplatin/S-1 with cisplatin/infusional fluorouracil in advancedgastric or gastroesophageal adenocarcinoma study: the FLAGS trial J Clin Oncol 2010 28 1547 1553 10.1200/JCO.2009.25.4706 20159816

[B29] Guimbaud R Louvet C Bonnetain F Viret F Samalin E Gornet JM André T Rebischung C Bouche O Jouve JL Final results of the intergroup FFCDGERCOR-FNCLCC 03–07 phase III study comparing two sequences of chemotherapy in advanced gastric cancers [abstract ] Ann Oncol 2010 21 8 viii 250

[B30] Kaya AO Coskun U Gumus M Dane F Ozkan M Isıkdogan A Alkis N Buyukberber S Yumuk F Budakoglu B Demirci U Berk V Bilici A Inal A Arpacı E Benekli M Anatolian Society of Medical Oncology (ASMO) The efficacy and toxicity of irinotecan with leucovorin and bolus and continuous infusional 5-fluorouracil (FOLFIRI) as salvage therapy for patients with advanced gastric cancer previously treated with platinum and taxane-based chemotherapy regimens J Chemother 2012 4 217 220 23040686 10.1179/1973947812Y.0000000020

